# Beyond the Petri Dish: The Performance of a Multiplex PCR-Based Pneumonia Panel in Hospitalized Patients With Lower Respiratory Tract Infection

**DOI:** 10.7759/cureus.105950

**Published:** 2026-03-27

**Authors:** Pooja Naharia, Anita Sharma, Puneet Bhatt, Akhil K Ravi, Aditya Joshi, Santosh Karade

**Affiliations:** 1 Department of Microbiology and Infectious Diseases, Army Hospital Research and Referral, Delhi, IND; 2 Department of Microbiology and Virology, Command Hospital, Udhampur, IND; 3 Department of Pulmonary and Critical Care Medicine, Military Hospital, Dehradun, IND; 4 Department of Anesthesiology and Critical Care Medicine, Army Hospital Research and Referral, Delhi, IND

**Keywords:** antimicrobial stewardship, biofire filmarray, coinfection, concordance, lower respiratory tract infection

## Abstract

Background

Lower respiratory tract infections (LRTIs) are a major cause of mortality in intensive care units (ICUs), necessitating rapid pathogen identification. This study evaluates the diagnostic performance of the multiplex polymerase chain reaction (PCR)‑based BioFire® FilmArray® Pneumonia Panel (BFPP) compared to conventional culture in hospitalized patients with LRTI at a tertiary care center.

Methodology

This retrospective observational study was carried out at a tertiary care hospital between January 2023 and June 2024. Bronchoalveolar lavage (BAL) culture and antibiotic sensitivity testing (ABST) data of individuals suspected to have LRTI were retrieved and compared to BFPP results for concordance.

Results

A total of 261 bronchoalveolar lavage (BAL) specimens were analyzed using the BFPP. Overall, BFPP detected 562 pathogens, comprising 419 bacterial isolates, 139 viral pathogens, and four atypical bacteria. Polymicrobial infections were identified in 33% of the specimens.

Among the antimicrobial resistance determinants detected, New Delhi metallo‑β‑lactamase (*NDM*) genes were the most prevalent (33%), followed by cefotaxime-Munich (*CTX‑M*)-like (25%) and oxacillinase-48 (*OXA‑48*)-like genes (18%). Concordance analysis between BFPP and conventional culture was feasible in 143 samples, demonstrating an overall agreement of 72%.

Using conventional culture as the reference standard, the BFPP assay showed a sensitivity of 90.2% and a specificity of 57.8%. The positive predictive value was 75.5%, while the negative predictive value was 80.5%. Notably, BFPP provided results significantly earlier than culture, with an average turnaround time advantage of approximately 52 hours.

Conclusion

BFPP improves detection rates of respiratory pathogens and shortens the turnaround time. The detection of antimicrobial resistance genes helps clinicians to initiate timely antibiotics and can play a pivotal role in antimicrobial stewardship.

## Introduction

Burden of respiratory infections

Globally, respiratory tract infections are the major cause of mortality and morbidity in all age groups and genders [1]. The lower respiratory tract includes the airway below the larynx, and infection manifests as fever, cough, nasal discharge, increased rate of breathing, chest retractions, and wheezing or crackles on auscultation [2]. Besides common bacteria, difficult-to-cultivate organisms such as atypical bacteria, viruses, and fungi are also important causes of lower respiratory tract infections (LRTIs). The early identification of the etiology of LRTI plays an important role in instituting goal-directed antimicrobial therapy, thereby preventing the development of antimicrobial resistance [3-5].

Conventional versus molecular methods for LRTI diagnosis

Conventional microbiological cultures (CMCs) require over 48 hours for identification and antimicrobial susceptibility testing from a respiratory sample. While awaiting those results, empirical broad-spectrum antibiotics are often prescribed. For a clinical microbiologist, the rapid identification of respiratory pathogens and their resistance mechanisms is a challenge. Early identification shortens the consumption of empirical broad-spectrum antibiotics and helps the effective implementation of the hospital antimicrobial stewardship program. Notably, molecular methods have been developed to supplement the time-consuming CMCs, for example, polymerase chain reaction (PCR)-based detection of bacterial genetic targets [6].

Role of the multiplex PCR panel

The commercially available BioFire® FilmArray® Pneumonia Panel (BFPP), from bioMérieux, has recently received FDA approval for the identification of multiple respiratory pathogens in a suspected case of pneumonia. This multiplex PCR-based assay can detect genomic targets of 18 bacteria, including three atypical bacteria and eight viruses that commonly cause pneumonia. In addition, this panel quantifies bacterial load in bronchoalveolar lavage (BAL)-like specimens and helps in the genotypic detection of seven selected antimicrobial resistance genes [7]. The BFPP assay takes 75 minutes (one hour and 15 minutes) and around two minutes for sample processing. This short turnaround time helps in the early initiation of targeted therapy. The utility of this panel needs to be investigated in the Indian setting. Thus, the primary objective of this study is to evaluate the performance of the BioFire FilmArray syndromic pneumonia panel-based approach with CMC in patients with LRTI admitted to the intensive care unit (ICU) of a tertiary care hospital.

## Materials and methods

Study design

This retrospective study was carried out at the microbiology laboratory of a tertiary care hospital after obtaining permission from the Institutional Ethics Committee of Army Hospital Research and Referral (approval protocol number: EC/NEW/INST/2023/3284). Microbiological data from the routine culture of BAL-like samples of individuals suspected to have LRTI processed between 1 January 2023 and 30 June 2024 were retrieved from the laboratory information system (LIS). The data from the pneumonia panel run through the BFPP assay during the same period were also retrieved. As per institutional policy, all lower respiratory samples are processed for conventional culture. However, only selected cases (critical or seriously ill) are subjected to the BioFire pneumonia panel assay due to cost constraints and also as indicated by the clinician in a critical care setting. Follow-up data were not recorded in the study.

Processing of respiratory samples

Respiratory samples were processed for multiplex molecular testing using the BioFire FilmArray system according to the manufacturer's instructions for the BioFire FilmArray Pneumonia Panel (BFPP). All procedures were performed under aseptic conditions within a class II biosafety cabinet. The single-use FilmArray pouch provided with the kit was loaded into the loading station, and the hydration solution was added to the designated hydration port. The specimen swab was mixed with the supplied sample buffer in the sample injection vial, which was subsequently inserted into the sample injection port of the pouch. The pouch barcode was scanned using the BioFire Torch system (bioMérieux, Marcy-l'Étoile, France), patient demographic details were entered, and the appropriate panel was selected for analysis. The BFPP assay was completed in approximately 75 minutes. Automated data analysis was performed by the FilmArray software (bioMérieux, Marcy-l'Étoile, France), and results were reported qualitatively as "detected" or "not detected" for each respiratory pathogen included in the panel. The assay also provides a semi-quantitative estimate of bacterial pathogens by analyzing nucleic acid copy numbers, typically reported in log_10_ bins (e.g., 10^5^/10^4^/10^3^ copies/mL).

Prior to culture processing, all samples were subjected to direct Gram staining. Only specimens demonstrating a predominance of inflammatory cells were selected for further microbiological analysis. Conventional culture was performed by inoculating samples onto blood agar, MacConkey agar, and chocolate agar plates, followed by incubation for 18-24 hours under appropriate conditions. Bacterial growth was evaluated based on Gram stain findings, colony morphology, and standard biochemical reactions. Commensal organisms (e.g., *Candida*), if isolated repeatedly on cultures, were taken as pathogenic and reported to the clinician. Definitive organism identification and antimicrobial susceptibility testing (AST) were carried out using the automated VITEK® 2 system (bioMérieux, Marcy-l'Étoile, France), in accordance with the manufacturer's guidelines. The turnaround time for the final microbiological report was obtained from the laboratory information system (LIS).

Analysis of results

The present study evaluated the diagnostic performance of the BioFire FilmArray Pneumonia Panel (BFPP) relative to conventional microbiological culture (CMC) in intensive care unit (ICU) patients with lower respiratory tract infections (LRTIs) at a tertiary care center. Furthermore, the study investigated BFPP-derived data on coinfections, antimicrobial resistance gene distribution, and the correlation between semi-quantitative pathogen load and culture positivity.

Samples with corresponding results from both the BFPP and CMCs were included for concordance analysis. Repeat samples from the same patient were excluded from analysis. Concordance was defined as the identification of the same organism by both methods (true positive). Discordant results were recorded when an organism was detected by conventional culture but not by BFPP or vice versa. Demographic and clinical variables were summarized using medians and proportions.

It is important to note that fungal pathogens are not included among the targets of the BioFire panel. Therefore, fungal organisms were identified only through conventional culture methods. As per standard clinical microbiology practice and existing guidelines, *Candida* species (spp.) isolated from respiratory specimens were interpreted as colonizers rather than true pathogens in the absence of supportive clinical, radiological, or microbiological evidence of invasive fungal disease.

## Results

**Figure 1:**
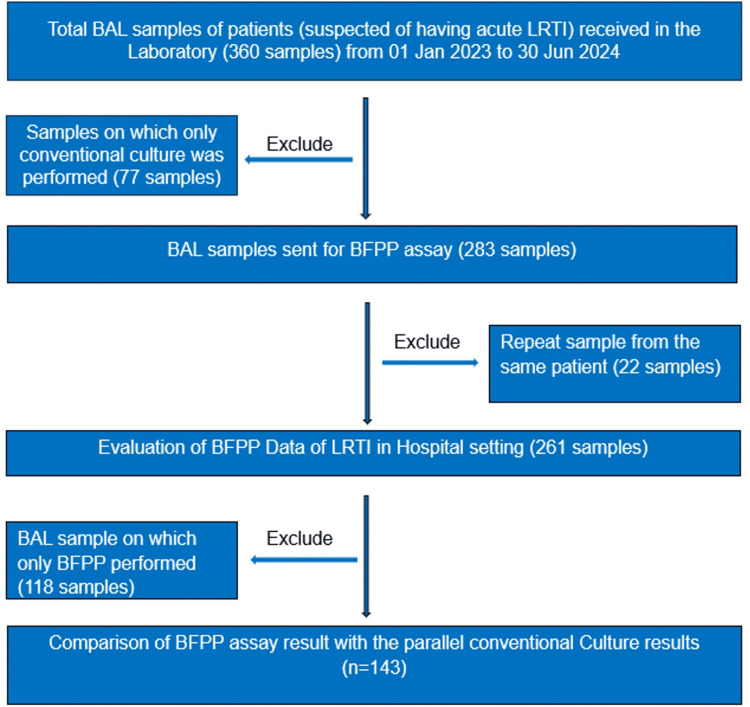
Flowchart showing study design BAL, bronchoalveolar lavage; LRTI, lower respiratory tract infection; BFPP, BioFire FilmArray Pneumonia Panel

During the 18‑month study period, a total of 360 bronchoalveolar lavage (BAL) samples were received in the laboratory for microbiological analysis. Out of these, 77 samples were excluded from the analysis as in these, only conventional culture was performed. The remaining 283 samples were processed for the BFPP assay. After the exclusion of repeat samples from the same patient (n = 22), 261 unique BAL samples were included for further analysis of the BFPP assay (Figure 1). Of these, 167 specimens were obtained from male patients. The mean age of the study population was 47.07 ± 24 years. The majority of samples were from patients aged 46-75 years (n = 137, 53%), followed by those aged <15 years (n = 40, 15%), 31-45 years (n = 40, 15%), >75 years (n = 24, 9%), and 16-30 years (n = 20, 8%). Most samples originated from critical care units (61%), including medical and surgical intensive care units and the high‑dependence unit, followed by the pediatric intensive care unit (12%), transplant intensive care unit (5%), and general wards.

Using the BioFire FilmArray Pneumonia Panel (BFPP), a total of 562 pathogens were detected across 261 BAL samples, comprising 419 bacterial isolates, 139 viral pathogens, and four atypical bacteria (*Mycoplasma pneumoniae* and *Legionella pneumophila*). No targets were detected in 38 samples (15%). Single‑pathogen infections were identified in 66 samples (25%), dual‑pathogen infections were identified in 70 samples (27%), and polymicrobial infections (>2 organisms) were observed in 87 samples (33%).

Among the typical bacterial pathogens, the most frequently identified organisms were *Acinetobacter calcoaceticus-baumannii* complex (22%), *Pseudomonas aeruginosa* (20%), and *Klebsiella pneumoniae* (20%). The distribution of bacterial pathogens and common antimicrobial resistance genes detected by BFPP is summarized in Table 1 and Table 2.

**Table 1:** Distribution of bacterial isolates identified by the BioFire FilmArray Pneumonia Panel (BFPP)

Typical bacteria	BFPP
*Acinetobacter* species (spp.)	94
Pseudomonas aeruginosa	83
Klebsiella pneumoniae	67
Escherichia coli	45
Haemophilus influenzae	37
Staphylococcus aureus	30
Streptococcus pneumoniae	18
Serratia marcescens	13
*Proteus* species	10
*Enterobacter cloacae* complex	9
Moraxella catarrhalis	7
Streptococcus agalactiae	4
Klebsiella oxytoca	1
Klebsiella aerogenes	1
Total	419

**Table 2:** Resistance gene targets detected by BFPP *NDM*, New Delhi metallo-ß-lactamase; *CTX-M*, cefotaxime-Munich; *OXA-48*, oxacillinase-48; *VIM*, Verona Integron-encoded metallo-β-lactamase; *KPC*, *Klebsiella pneumoniae* carbapenemase; *IMP*, imipenemase metallo-β-lactamase; *mecA/C* and *MREJ*, methicillinase (mec) and staphylococcal cassette chromosome mec (SCC mec)-orfX right-extremity junction

Resistance genes	Percentage
NDM	33%
CTX-M	25%
OXA-48	18%
VIM	14%
KPC	4%
IMP	3%
*mecA/C* and *MREJ*	3%

Among viral pathogens, human rhinovirus/enterovirus was the most frequently detected (33%), followed by influenza A virus (24%), respiratory syncytial virus (13%), and coronavirus (12%). The distribution of other respiratory viruses detected by BFPP is presented in Table 3. Of the atypical bacteria, *Mycoplasma pneumoniae* and *Legionella pneumophila* were each detected in two BAL specimens.

**Table 3:** Distribution of respiratory viruses detected by BioFire FilmArray Pneumonia Panel (BFPP) in BAL specimen BAL: bronchoalveolar lavage

Virus identified	Total
Human rhinovirus/enterovirus	46 (33%)
Influenza A	33 (24%)
Respiratory syncytial virus	18 (13%)
Coronavirus	17 (12%)
Human metapneumovirus	7 (5%)
Parainfluenza virus	11 (8%)
Influenza B	4 (3%)
Adenovirus	3 (2%)

During the conventional culture processing of BAL samples, *Candida* species were isolated in a limited number of cases. In our cohort, no case demonstrated repeated isolation of *Candida* species or accompanying features suggestive of invasive candidiasis, and hence, these isolates were excluded from final fungal pathogen analysis. Only one non-*Candida* fungal isolate met the predefined criteria for clinical significance and was therefore reported as a true fungal pathogen.

Out of the 261 BAL samples processed for the BFPP assay, concordance analysis between the BFPP and conventional culture was feasible only in 143 BAL specimens, as in these samples only, both BFPP and conventional culture were processed.

Concordance analysis between the BFPP and conventional culture was feasible in 143 BAL specimens for which results from both diagnostic modalities were available. Conventional culture yielded positive results in 86 samples, with the most frequently isolated organisms being *Acinetobacter calcoaceticus-baumannii* complex (42%), *Klebsiella pneumoniae* (21%), and *Pseudomonas aeruginosa* (20%). Polymicrobial growth (≥2 organisms) was observed in four samples. The distribution of bacterial isolates identified by conventional culture is summarized in Table 4.

**Table 4:** Distribution of bacterial isolates identified by conventional culture

Organism	Culture
*Acinetobacter* species (spp.)	36
Pseudomonas aeruginosa	17
Klebsiella pneumoniae	18
Escherichia coli	3
Burkholderia cepacia	3
Staphylococcus aureus	1
*Proteus* species	1
Elizabethkingia meningoseptica	1
Enterococcus faecalis	1
Aspergillus fumigatus	1
Mixed growth	4
Total	86

**Figure 2:**
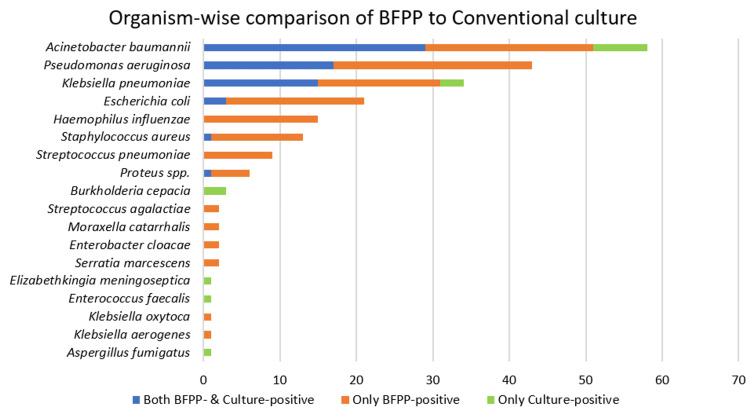
Organism-wise comparison of BFPP to conventional culture BFPP, BioFire FilmArray Pneumonia Panel; spp., species

Overall concordance between BFPP and culture for bacterial identification (excluding viral and atypical pathogens) was observed in 72% of samples, while discordant results were noted in 28%. Among the 143 samples analyzed, both BFPP and culture were positive in 53% (n = 74), and both methods were negative in 24% (n = 33). Detailed comparative results are presented in Figure 2 and Table 5. The BFPP‑negative, culture‑positive results were largely attributable to organisms not represented in the BFPP pneumonia panel, which includes *Burkholderia cepacia*, *Elizabethkingia meningoseptica*, *Enterococcus faecalis*, and fungal isolates. Samples demonstrating mixed growth on culture were excluded from this comparative analysis.

**Table 5:** Organism-wise comparison between the conventional culture and the BFPP BFPP, BioFire FilmArray Pneumonia Panel; spp., species

Organism identified	Both BFPP- and culture-positive	Only BFPP-positive	Only culture-positive
*Acinetobacter baumannii*	31	15	6
*Pseudomonas aeruginosa*	17	24	0
*Klebsiella pneumoniae*	15	15	2
*Escherichia coli*	3	18	0
*Haemophilus influenzae*	0	15	0
*Staphylococcus aureus*	1	12	0
*Streptococcus pneumoniae*	0	9	0
*Proteus* spp.	1	5	0
*Burkholderia cepacia*	0	0	3
*Streptococcus agalactiae*	0	2	0
*Moraxella catarrhalis*	0	2	0
*Enterobacter cloacae*	0	2	0
*Serratia marcescens*	0	2	0
*Elizabethkingia meningoseptica*	0	0	1
*Enterococcus faecalis*	0	0	1
*Klebsiella oxytoca*	0	1	0
*Klebsiella aerogenes*	0	1	0
*Aspergillus fumigatus*	0	0	1

The BFPP assay also provides semi‑quantitative pathogen load estimates expressed as bin‑based copies/mL values. A positive correlation was observed between BFPP semi‑quantitative values and culture positivity. BFPP values of ≥10^7^ copies/mL were associated with a 73% culture positivity rate, whereas organisms detected at 10^6^ copies/mL were successfully isolated by culture in 31% of cases. When multiple bacterial pathogens were detected, the actual pathogen was decided based on clinical evaluation and a bin value of over 10^4^ copies/mL.

The mean turnaround time from sample collection to the availability of antibiotic susceptibility results for conventional culture was 54.55 hours, compared with a mean time of 2.16 hours for BFPP results in the same set of samples.

## Discussion

Role of point-of-care tests in the diagnosis of LRTI

LRTIs significantly contribute to morbidity, mortality, and economic burden in acute healthcare settings [8]. Early diagnosis and targeted treatment can prevent complications and the emergence of drug resistance. However, conventional cultures are time-consuming and labor-intensive. With the introduction of point-of-care tests such as BFPP assays, the diagnosis of causative agents and implicated resistance genes can be achieved within one and a half hours. This study assessed the utility of BFPP in the laboratory diagnosis of suspected LRTI in an acute healthcare setting.

The age distribution of the 261 unique BAL samples highlights a predominance of middle-aged and older adults, with over half (53%) from the 46-75-year age group, reflecting the typical demographic burden of severe respiratory conditions requiring BAL in critical care settings. A notable proportion (15%) came from young children under 15 years, likely driven by pediatric intensive care cases, while younger adults (16-30 years, 8%; 31-45 years, 15%) and the elderly (>75 years, 9%) were less represented.

Male preponderances in hospitalization for LRTI in our study corroborated with other studies [9]. As seen in previous studies, our study also showed a higher bacterial positivity rate with BFPP (73%) compared to the conventional cultures (60%) [10-12]. Bacterial etiology was a more common cause of LRTI as compared to viral [13]. Most common bacterial isolates identified by the BFPP assay reflected the organisms prevalent in the hospital care setting, such as *Acinetobacter baumannii*, *Pseudomonas aeruginosa*, *Klebsiella pneumoniae*, *Escherichia coli*, and *Staphylococcus aureus* [14]. The BFPP assay could identify the viral etiology and the atypical organisms (*Mycoplasma pneumoniae *and *Legionella pneumophila*), which may be difficult to isolate in a peripheral setting by conventional culture methods. The unnecessary use of broad-spectrum antibiotics was curtailed in cases suggestive of viral etiology by BFPP.

The early identification of the resistance gene of the organism by BFPP guided clinicians to start appropriate antibiotics. The emergence and spread of extended-spectrum beta-lactamases (ESBLs)-mediated resistance to cephalosporins among Gram-negative bacterial isolates in hospitals, as well as in community settings, is a cause for concern worldwide. Prior studies from India reported the prevalence of ESBL between 60% and 80% in Gram-negative isolates [15]. The most common resistance gene identified in this study was *NDM* (33%), followed by *CTX-M* (25%) and *OXA-48* (18%).

In our study, 30 cases of *Staphylococcus aureus* were identified using BFPP; however, only one could be isolated through culture methods, indicating the reduced sensitivity of conventional culture. Among these, BFPP successfully detected 14 cases of methicillin-resistant *Staphylococcus aureus* (MRSA), aiding clinicians in implementing timely infection control measures, even when *Staphylococcus aureus* was not isolated by conventional culture. The early identification of resistance genes, such as methicillinase (mec) and staphylococcal cassette chromosome mec (SCC mec)-orfX right-extremity junction (*mecA* and *MREJ*), has been associated with reduced patient stays in critical care wards and lower mortality rates [16,17]. However, when multiple organisms are identified (*E. coli*, *Klebsiella*, or *Acinetobacter*), along with multiple resistance genes (*NDM* + *CTX-M* + *OXA-48*), BFPP cannot distinguish which organism is implicated for different resistance genes.

The BFPP assay is user-friendly with a short turnaround time of about 90-100 minutes, including the sample processing time. Reporting by conventional bacterial cultures, along with the antibiotic sensitivity testing (ABST), takes around 48-56 hours. Also, the manual culture method is labor-intensive and requires trained manpower and quality control of media, incubators, antibiotic discs, etc. In our study, a lead time of approximately 50 hours was obtained by BFPP, crucial for decision-making in ICUs.

The study identified multiple respiratory pathogens in 33% of the specimens, with coinfection (bacterial and viral) detected in 35% of the samples. The relevance of coinfection, especially in patients on long-term ventilators, is arguable. The coinfecting bacteria may be an innocent bystander or hospital contaminant harboring a resistant gene. Multiplex PCR is a highly sensitive assay, and preanalytical variables such as sample quality, sample source, the amount of target, and the time of sampling may affect the result. If the treatment is started based on the commensal flora, it can be harmful to the patient and may also enhance the development of antibiotic resistance [18]. Thus, the interpretation of BFPP results needs to be made considering the clinical condition of patients and biomarkers of sepsis.

Comparison of the BFPP assay on BAL samples to conventional microbiological cultures

The BFPP assay on BAL samples was compared to the results of the conventional culture, which is the gold standard in the isolation of the causal organism. To rule out the preanalytical errors in the processing of the samples and also to prevent the reporting of oral commensals and colonizers in the conventional cultures, Gram staining on the direct samples was checked before reporting on the conventional cultures. Hence, only 143 could be evaluated for concordance between BFPP and conventional cultures, as data on parallel cultures were available only on these samples. Concordance was found only in 72% of the samples.

The sensitivity of the BFPP assay, keeping conventional cultures as the gold standard, was 90.2% and specificity 57.8%. The positive predictive value of BFPP was 75.5% and the negative predictive value 80.5%. The sensitivity of the BioFire pneumonia panel is higher than the conventional cultures, and specificity is low, which may be attributed to the fastidious nature of some organisms that could not be cultivated on conventional cultures. Because of higher sensitivity, the assay can be utilized in a critical care setting for the early start of appropriate treatment.

Reasons for discordant results by PCR and culture

In the present study, discordant results between the BioFire FilmArray Pneumonia Panel (BFPP) and conventional culture were observed in 28% of the samples. Several factors may account for this discrepancy. First, certain organisms recovered by conventional culture are not included among the BFPP target pathogens, such as *Burkholderia cepacia* and *Elizabethkingia meningoseptica*, resulting in culture‑positive but BFPP‑negative findings.

Second, BFPP is a multiplex PCR‑based assay that detects microbial nucleic acids (DNA/RNA), enabling the identification of non-viable organisms, colonizers, or commensals that may not be clinically significant or recoverable by culture. In contrast, organisms present in low quantities, involved in polymicrobial infections, or overgrown by normal respiratory flora may be missed during conventional culture processing, leading to discordant results.

Third, fastidious organisms such as *Haemophilus influenzae* may fail to grow under standard culture conditions, be overgrown by competing flora, or lose viability during transport and processing, whereas their genetic material may still be detected by BFPP.

Prior empirical antimicrobial therapy represents another important contributor to discordance. Antibiotic exposure before sample collection can suppress bacterial viability and inhibit growth in culture, while BFPP may still detect residual nucleic acids from non-viable organisms. In the present study, organisms such as *Enterobacter cloacae*, *Streptococcus agalactiae*, and *Streptococcus pneumoniae* were detected by BFPP at low copy numbers but failed to grow in culture.

The PCR-based BFPP is a highly sensitive assay; however, it cannot differentiate from innocuous colonization from actual pathogen, especially in patients on prolonged ventilatory support. The apparent low specificity is partly due to culture false negatives. Therefore, conventional culture may be an imperfect gold standard when compared with a PCR-based assay in our study.

Additionally, fungal pathogens, including *Aspergillus fumigatus*, were isolated by culture but not detected by BFPP, as fungal targets are not incorporated in the current pneumonia panel. The identification of fungal pathogens, particularly molds such as *Mucor* and *Aspergillus* species, is clinically important in critical care settings, where they represent significant causes of lower respiratory tract infections, especially among immunocompromised patients.

The current BFPP assay primarily targets pathogens associated with community‑acquired pneumonia. These findings highlight the need for the expansion and customization of respiratory pathogen panels based on geographical prevalence and clinical setting (hospital‑acquired versus community‑acquired infections). Pathogens commonly implicated in healthcare‑associated LRTIs, such as *Morganella morganii*, *Citrobacter freundii*, *Stenotrophomonas maltophilia*, *Enterococcus* species, and fungal pathogens including *Candida*, *Mucor*, and *Aspergillus* species, warrant consideration for inclusion.

In recent years, the BioFire technology has undergone significant advancements, including the expansion of target panels and increased system throughput with the BioFire Torch platform, which now supports up to 12 modules operating simultaneously. The introduction of the BioFire SPOTFIRE system represents a further evolution toward point‑of‑care diagnostics, providing rapid results within approximately 15 minutes for selected syndromic panels, including respiratory and sore throat infections [19]. The key features of the BFPP assay from this study are listed in Table 6, and Table 7 compares our findings with previous studies.

**Table 6:** Salient features of BioFire FilmArray Pneumonia Panel (BFPP)

Benefits	Challenges
Short turnaround time, user-friendly, and less labor-intensive	The high initial cost of the instrument and the recurring cost of consumables limit its use in public healthcare
Compact instrument. Closed system; hence, chances of contamination are rare	Limited number of targets
The assay has wide coverage, including the fastidious organisms, atypical organisms, and viruses	Fungal respiratory pathogens not included in the panel
Provide a semi-quantitative estimation of the burden of respiratory pathogens	Identifies non-viable organisms also, giving false positive results
Identifies the implicated resistance genes also, thereby promoting antimicrobial stewardship	Whenever multiple organisms are detected by BFPP, the resistance gene identified cannot be implicated to a single organism
Highly sensitive assay	Cannot distinguish the colonizer from the actual pathogen

**Table 7:** Review of prior studies on the diagnostic utility of BioFire FilmArray Pneumonia Panel BFPP, BioFire FilmArray Pneumonia Panel; PN, pneumonia

Study	Sample size	Study location	Year	Salient points
Van Der Westhuyzen et al. [11]	125	Cape Town, South Africa	2023	Positivity rate, 86.6%; positive percent agreement (PPA), 100%; negative percent agreement (NPA), 88%; coinfection, 20%
Moy et al. [12]	-	-	2023	Overall sensitivity and specificity, respectively, 94% and 98%
Ginocchio et al. [20]	2463	Multinational	2021	Concordance, 49.10%; positivity rate, 76.13%
Buchan et al.[10]	259	USA	2020	Concordance, 93.2%; PPA, 96.2%; NPA, 98.1%. PN panel resulted in a 63.3% increase in specimens reported as positive and a 94.8% increase in the total number of bacterial targets detected
Lee et al.[21]	59	Taiwan	2019	PPA, 90%; NPA, 97.4%. Concordance rate of 53.6% for the culture-positive specimens and 86.3% for the culture-negative specimens. Coinfection: 42.3%
Present study	261	Delhi, India	2024	Bacterial positivity rate, BFPP (73%) and culture (60%); coinfection, 35%; concordance, 72%. Sensitivity compared to conventional cultures was 90.2% and specificity 57.8%. Positive predictive value was 75.5% and negative predictive value 80.5%

The findings of this study should be interpreted in light of certain limitations, including the relatively small sample size, retrospective study design, and data derived from a single center. Additionally, the high cost of consumables limits the routine use of the BioFire FilmArray Pneumonia Panel in public healthcare settings; consequently, its application in this study was largely restricted to critically ill patients.

## Conclusions

In conclusion, the importance of conventional cultures cannot be ignored, as some of the critical pathogens in the ICU setting are not available in the panel targets. However, the implementation of the BioFire FilmArray Pneumonia Panel in acute healthcare settings significantly reduces the turnaround time for microbiological diagnosis. The assay demonstrates higher sensitivity compared to conventional culture methods and enables the rapid identification of viral pathogens, atypical organisms, and polymicrobial infections. Additionally, the timely detection of relevant antimicrobial resistance genes by BFPP can support the early optimization of antimicrobial therapy and strengthen hospital antimicrobial stewardship programs.
